# Probabilistic classification of anti‐SARS‐CoV‐2 antibody responses improves seroprevalence estimates

**DOI:** 10.1002/cti2.1379

**Published:** 2022-03-02

**Authors:** Xaquin Castro Dopico, Sandra Muschiol, Nastasiya F Grinberg, Soo Aleman, Daniel J Sheward, Leo Hanke, Marcus Ahl, Linnea Vikström, Mattias Forsell, Jonathan M Coquet, Gerald McInerney, Joakim Dillner, Gordana Bogdanovic, Ben Murrell, Jan Albert, Chris Wallace, Gunilla B Karlsson Hedestam

**Affiliations:** ^1^ 27106 Department of Microbiology, Tumor and Cell Biology Karolinska Institutet Stockholm Sweden; ^2^ 59562 Department of Clinical Microbiology Karolinska University Hospital Stockholm Sweden; ^3^ 2152 Cambridge Institute of Therapeutic Immunology & Infectious Disease University of Cambridge Cambridge UK; ^4^ 59562 Department of Infectious Diseases Karolinska University Hospital Huddinge Sweden; ^5^ 8075 Department of Clinical Microbiology Umeå Universitet Umeå Sweden; ^6^ 27106 Division of Pathology Department of Laboratory Medicine Karolinska Institutet Huddinge Sweden; ^7^ 2152 Medical Research Council Biostatistics Unit University of Cambridge Cambridge UK

**Keywords:** antibody responses, antibody testing, COVID‐19, probability, SARS‐CoV‐2, serology

## Abstract

**Objectives:**

Population‐level measures of seropositivity are critical for understanding the epidemiology of an emerging pathogen, yet most antibody tests apply a strict cutoff for seropositivity that is not learnt in a data‐driven manner, leading to uncertainty when classifying low‐titer responses. To improve upon this, we evaluated cutoff‐independent methods for their ability to assign likelihood of SARS‐CoV‐2 seropositivity to individual samples.

**Methods:**

Using robust ELISAs based on SARS‐CoV‐2 spike (S) and the receptor‐binding domain (RBD), we profiled antibody responses in a group of SARS‐CoV‐2 PCR+ individuals (*n* = 138). Using these data, we trained probabilistic learners to assign likelihood of seropositivity to test samples of unknown serostatus (*n* = 5100), identifying a support vector machines‐linear discriminant analysis learner (SVM‐LDA) suited for this purpose.

**Results:**

In the training data from confirmed ancestral SARS‐CoV‐2 infections, 99% of participants had detectable anti‐S and ‐RBD IgG in the circulation, with titers differing > 1000‐fold between persons. In data of otherwise healthy individuals, 7.2% (*n = *367) of samples were of uncertain serostatus, with values in the range of 3‐6SD from the mean of pre‐pandemic negative controls (*n* = 595). In contrast, SVM‐LDA classified 6.4% (*n* = 328) of test samples as having a high likelihood (> 99% chance) of past infection, 4.5% (*n* = 230) to have a 50–99% likelihood, and 4.0% (*n* = 203) to have a 10–49% likelihood. As different probabilistic approaches were more consistent with each other than conventional SD‐based methods, such tools allow for more statistically‐sound seropositivity estimates in large cohorts.

**Conclusion:**

Probabilistic antibody testing frameworks can improve seropositivity estimates in populations with large titer variability.

## Graphical abstract

Correctly classifying low‐titer antibody responses is challenging using conventional assay cutoffs. To address this issue, we trained suitable probabilistic learners to assign likelihood of seropositivity. These more quantitative methods improve seroprevalence estimates and have potential application to the clinical setting.

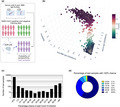

## Introduction

The SARS‐CoV‐2 pandemic has illustrated the importance of antibody (Ab) testing, clinically, epidemiologically, and to guide public health measures.^1^ However, a common problem for Ab tests is the correct classification of seropositivity as negative control values overlap true positive low‐titer values, impacting estimates of past infection.

Most Ab tests apply a strict cutoff to determine seropositivity,^2^, ^3^, ^4^ such as the ratio between known positive and negative serum calibrators, or 3 or 6 standard deviations (SD) from the mean of negative controls. This is a conservative approach that tries to avoid misclassifying any true negatives as positive, although it may as a result miss a proportion of true positives, and through a statistically costly dichotomization, leads to a loss of information. Although 6 SD often serves well for an assay to achieve high specificity, the metric is not learnt in any formal data‐driven manner and is highly dependent on the nature and number of negative control samples used to determine the cutoff, which have their own variability. Indeed, the optimal cutoff may depend upon use of the assay. A 6 SD threshold may favor specificity, whilst a 3 SD threshold is likely to be more sensitive. This dependence on a threshold is problematic because, while some samples with extremely high or low readouts and are easily classifiable, many measurements may be close to the threshold and their classification changes according to small changes in the threshold, for example, a different assay batch, serum calibrator, or low numbers of negative controls.

This is especially pertinent for SARS‐CoV‐2, as most infections follow a mild clinical course that typically engenders lower Ab titers than do infections characterized by severe disease.^5^, ^6^, ^7^, ^8^, ^9^ A similarly wide range of Ab titers has been observed after COVID‐19 vaccination.^10^, ^11^, ^12^ As Ab responses decline from peak levels over time, this implies that most previously infected/vaccinated persons will eventually have Ab titers difficult to detect and classify, in the absence of re‐infection/vaccination. As many common Ab tests used in clinical, research, and public health practice have low sensitivity (e.g. lateral flow tests) compared to laboratory‐based methods, the problem is compounded.

The goal of a serology study could be to estimate the seroprevalence in a sampled population. We argue that this goal is better achieved by using a probabilistic classification framework, which estimates the probability (% chance) that a sample is *positive* or *negative*, rather than the individual binary classification supported by SD‐based cutoffs. We reasoned that cutoff‐independent frameworks trained on real‐world data would better consider the wide range of the response and resolve some of this uncertainty. To this end, we first developed highly sensitive and specific anti‐SARS‐CoV‐2‐Spike and ‐RBD ELISA assays based on native‐like antigens, and then used them in tandem to explore how isotype‐level anti‐SARS‐CoV‐2 Ab responses varied with disease severity and duration; in *n* = 138 RT‐PCR‐confirmed COVID‐19 cases distributed across the clinical spectrum. The IgG information, and the responses from a large number (*n* = 595) of pre‐pandemic negative controls, were then used to train suitable probabilistic learners to assign likelihood of seropositivity to test samples of unknown serostatus, in this case samples collected from blood donors and pregnant women (*n* = 5100) during community transmission in Stockholm, Sweden throughout 2020 and early 2021.^13^

We chose to study spike (S)‐directed Abs as these are robustly induced by SARS‐CoV‐2 infection at the population level (e.g. IgG is estimated to be present in > 91.1% of RT‐PCR‐positive adult cases after infection with the ancestral strain^14^), and a subset of S‐directed antibodies (primarily RBD binders) mediate virus neutralizing activity.^14^, ^15^ Indeed, all currently‐approved COVID‐19 vaccines are based on S, with the aim of inducing neutralizing Abs that block viral entry into ACE2‐expressing target cells.^11^, ^16^ Therefore, anti‐S and ‐RBD responses are also the best available correlates of vaccine protection. As both infection‐ and vaccination‐induced anti‐S titers vary between individuals and decline with time, probabilistic methods to determine seropositivity could improve clinical and epidemiological investigations.

## Results

Study samples are detailed in Table 1 and Supplementary table 1.

**Table 1:** Study samples

Sample groups	Sample numbers, age ranges and collection dates
SARS‐CoV‐2 RT‐PCR+ individuals^a^	*n* = 105
Females	44 (41.9%)
Males	61 (58.1%)
Age range (years)	18–80
Mean age (years)	
Females	53.0
Males	55.0
Non‐hospitalized (Category 1)	*n* = 53
Females, Males (mean age, years)	28, 25 (51.5)
Hospitalized (Category 2)	*n* = 31
Females, Males (mean age, years)	12, 17 (54.4)
Intensive care (Category 3)	*n* = 21
Females, Males (mean age, years)	3, 17 (60.4)
Sample collection	March–May 2020
SARS‐CoV‐2 RT‐PCR+ hospital employees^b^	*n* = 33
Sample collection	July 2020
Vaccinated individuals^b^	*n* = 30
Sample collection	April–October 2021
Blood donors^b^	*n* = 2600
Sample collection	March 2020–January 2021
Pregnant women^b^	*n* = 2500
Sample collection	March 2020–January 2021
Historical (pre‐pandemic) blood donors^b^	*n* = 595
Sample collection	March–June 2019
Endemic CoV+ donors^b^	*n* = 20
Sample collection	July–December 2019
Sample subset used for assay development	
Pre‐pandemic controls	*n* = 100
RT‐PCR+ individuals (random subset^a^)	*n* = 38
Blood donor samples (March)	*n* = 100
Endemic coronavirus RT‐PCR+ donors	*n* = 20

^a^ Individuals under the care of Karolinska University Hospital.

^b^ No additional metadata was available.

### SARS‐CoV‐2 Ab test development

Highly sensitive and specific ELISA assays to profile IgM, IgG and IgA responses against ancestral SARS‐CoV‐2 pre‐fusion‐stabilized spike (S) glycoprotein trimers, the RBD, or the nucleocapsid (N), were developed alongside a diagnostic clinical laboratory responsible for monitoring seroprevalence during the pandemic. Samples were collected between March 2020 and January 2021, and therefore represent responses to the ancestral SARS‐CoV‐2 strain. The spike trimers^17^ were produced in‐house and their quality was confirmed by cryo‐EM.^18^ A representative subset of study samples was used for assay development (Table 1), and we did not observe reproducible IgG reactivity to S or RBD across the 595 historical negative controls collected before the pandemic.

Responses to S and the RBD were highly correlated in RT‐PCR‐confirmed COVID‐19 cases (*n* = 138), and our assay revealed a greater than 1000‐fold inter‐personal differences in anti‐viral IgG titers between Ab‐positive individuals when examining serially diluted sera. One individual had detectable anti‐nucleocapsid IgG at 1:200 000 serum dilution (Supplementary figure 1a). In RT‐PCR+ individuals, anti‐viral IgG titers were comparable for S (EC_50_ = 3064; 95% CI [1197–3626]) and N (EC_50_ = 2945; 95% CI [543–3936]) and slightly lower for RBD [EC_50_ = 1751; 95% CI 966–1595]. We noted that a subset (*~*10%) of SARS‐CoV‐2‐confirmed individuals did not have detectable IgG responses against the SARS‐CoV‐2 nucleocapsid protein (Supplementary figure 1a), as previously reported.^19^ Therefore, we did not explore responses to the nucleocapsid protein further in this study.

### Anti‐spike Ab titers and neutralizing responses are positively correlated with disease severity

We initially examined Ab responses in relation to clinical severity using a 6 SD assay cutoff. To arrive at this cutoff, we repeatedly analyzed a large number (*n* = 595) of SARS‐CoV‐2‐negative controls (pre‐pandemic blood donor samples collected during the Spring of 2019) alongside test samples throughout the study. This number enabled us to generate accurate estimates of mean values and their variation in a non‐exposed population.

**Figure 1:**
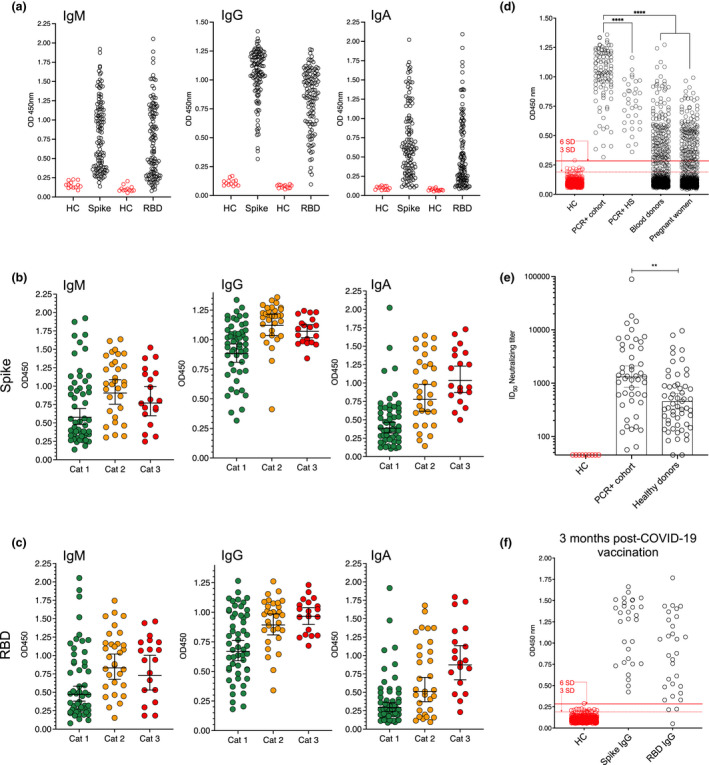
Anti‐SARS‐CoV‐2 Ab responses in RT‐PCR+ and vaccinated individuals are spread over a wide titer range. **(a)** IgM, IgG and IgA anti‐S and –RBD responses in individuals RT‐PCR+ for SARS‐CoV‐2 RNA (COVID‐19 patients and hospital staff, *n* = 138). A small number of healthy controls (HC, pre‐pandemic samples) are shown for each assay and isotype. **(b)** Anti‐S isotype‐level responses according to COVID‐19 clinical status. Cat 1: mild/asymptomatic. Cat 2: hospitalized. Cat 3: Intensive care. **(c)** Anti‐RBD isotype‐level responses according to COVID‐19 clinical status. **(d)** Anti‐S responses in RT‐PCR+ cases (*n* = 105), RT‐PCR+ hospital staff (HS, *n* = 33), blood donor (*n* = 1000) and pregnant women (*n* = 1000) serum samples collected during the first three months of the pandemic. 3 and 6 SD cutoffs calculated from *n = *595 historical control samples are shown by dashed and solid red lines, respectively. **(e)** Neutralizing ID_50_ titers in RT‐PCR+ individuals and a subset of healthy donors (*n* = 56) collected during the first three months of the pandemic. **(f)** Anti‐S and ‐RBD IgG responses 3 months post‐boost in individuals vaccinated with either BNT162b2 (*n* = 10), mRNA‐1273 (*n* = 10) or ChAdOx1 (*n* = 10) COVID‐19 vaccines. 3 and 6 SD cutoffs are shown by dashed and solid red lines, respectively. Error bars represent the geometric mean with 95% CIs.

Using this 6 SD cutoff, we detected potent IgG responses against S and RBD in 100% and 99% of RT‐PCR‐confirmed infections (*n* = 138), respectively, supporting that natural infection with ancestral SARS‐CoV‐2 provoked a robust B cell response in the majority of cases, as reported.^20^, ^21^ IgM and IgA responses were generally weaker, more variable between individuals, and spread over a larger titer range (Figure 1a).

To examine isotype‐level responses further, RT‐PCR+ COVID‐19 patients (*n* = 105, excluding Karolinska University Hospital employees) were grouped according to their clinical status, as non‐hospitalized (Category 1, mild/asymptomatic; 49.5% of total), hospitalized (Category 2; 29.5%), or intensive care patients (Category 3; 20%). To validate our clinical classification, we measured serum IL‐6 levels in a random subset of RT‐PCR+ individuals from each of the 3 categories (*n* = 64) and found that these were particularly high in Category 3 (intensive care) individuals (Supplementary figure 1b).

Multivariate analysis, accounting for the effects of age, sex and days from symptom onset/date of RT‐PCR test, revealed that increased anti‐viral IgM, IgG and IgA titers were associated with disease severity (Figure 1b and c, Supplementary table 2) within the three months of infection/RT‐PCR date. IgA isotypes were most strongly associated with worsening disease, although IgM and IgA titers declined with time from symptom onset, in agreement with their t½ in the circulation after viral clearance (Supplementary table 2). Taken together, these results support that anti‐S and ‐RBD IgG responses are better indicators of past SARS‐CoV‐2 infection at the population level than are IgM or IgA, or anti‐nucleocapsid Abs.

We next characterized the *in vitro* virus neutralizing antibody response of RT‐PCR+ individuals using an established pseudotyped virus neutralization assay^13^, ^18^, ^22^ and detected neutralizing antibodies in the serum of all SARS‐CoV‐2 RT‐PCR+ individuals screened (*n* = 48), while neutralizing responses were not seen in samples before seroconversion or negative controls (ID_50_ cutoff = 45) (Supplementary figure 1c). As observed in the binding assay, a large range of neutralizing ID_50_ titers was apparent in the samples, with binding and virus neutralization being highly correlated (Supplementary table 2). In agreement with the binding data, the strongest neutralizing responses were observed in samples from patients who required intensive care, Category 3 (g.mean ID_50_ = 5058; 95% CI [2422–10 564]). Mild infections, Category 1, had ID_50_ neutralization titers averaging about 770, similar to first‐generation COVID‐19 vaccines.^11^

### Anti‐SARS‐CoV‐2 Ab responses in healthy individuals of unknown serostatus

Compared to RT‐PCR+ samples collected within the first 63 days of symptom onset, anti‐viral Ab titers were lower in seropositive samples from otherwise healthy blood donors and pregnant women collected during the first three months of community transmission in Stockholm. Titers in healthy donor samples were similar to those observed in RT‐PCR+ hospital employees (*n* = 33) also sampled within three months post‐infection who were not hospitalized for COVID‐19 (Figure 1d). The same trend between RT‐PCR+ and healthy donor samples (collected in the first 3 months of the pandemic) was observed in terms of neutralizing Ab responses (Figure 1e).

Furthermore, although we observed generally robust anti‐Spike IgG responses after two doses of COVID‐19 vaccines (BNT162b2, mRNA‐1273 or ChAdOx‐1, *n* *=* *30 in total*), these were also spread over a large titer range (Figure 1f, Supplementary table 3). According to a 6SD cutoff, 10% of the individuals surveyed did not have detectable RBD responses by 3 months post‐dose two, while a further 10% of RBD measurements were below 9 standard deviations from the mean of negative controls. Therefore, as vaccine‐induced titers have been reported to decline over time,^23^, ^24^, ^25^ a greater proportion of vaccinated individuals are predicted to have responses that are difficult to classify by 6, 9 or 12 months‐post vaccination.

**Figure 2:**
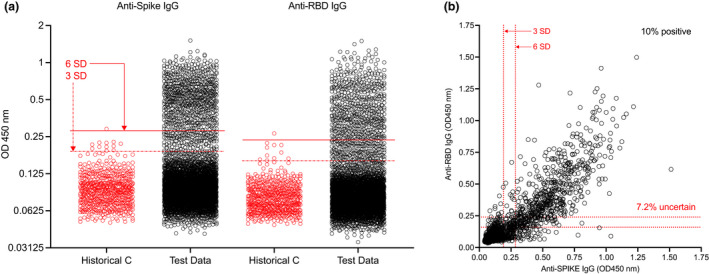
Low‐titer responses are difficult to classify using conventional assay thresholds. **(a)** Anti‐S and RBD‐ IgG responses in pre‐pandemic negative controls (*n* = 595) and blood donor and pregnant women test data (*n* = 5100). 3 and 6 SD cutoffs based on all negative control values are shown by dashed and solid red lines, respectively. **(b)** Anti‐S vs. ‐RBD IgG responses. 10% of samples were seropositive against both antigens at 6 SD, while 7.2% of values were of uncertain serostatus, depending on the assay and cutoff used.

As expected, given the predominance of mild infections and greater cohort heterogeneity at the population level, many values for blood donor and pregnant women samples (from *n* = 5100) scored close to or between conventional 3 and 6 SD cutoffs for the S or RBD assays (Figure 2a), complicating their classification using a hard boundary. There is no single standard multiple for SD‐based thresholds, which typically range from 3 to 6 SD above the mean observed in negative controls, and it is not clear how to combine inferences when responses to different antigens are measured simultaneously. For example, we found 6.5% healthy individual samples to be classified differently by anti‐S or anti‐RBD IgG responses, and a further 0.7% to be classified differently depending on whether a 3 SD or 6 SD cutoff was chosen (Figure 2b). We concluded that binary calls could not adequately combine information from the two antigens nor represent the uncertainty in classification for points near SD‐based cutoffs, where OD values overlap for true seronegative and seropositive samples.

### Probabilistic tools for estimating SARS‐CoV‐2 seropositivity

The 3 SD or 6 SD classifiers learn their positivity thresholds using training data from known negative samples. However, more flexible alternatives exist which can exploit information from both known negative and positive samples and provide not just a binary seropositive or seronegative call, but an estimate of the probability that a sample is from a seropositive individual, which can capture uncertainty in inference where appropriate. They are also adapted to learn from multiple measures simultaneously and can exploit correlation between them, as exists here between anti‐S and anti‐RBD. They can be used for the same purposes as binary classifiers, albeit with adapted methodology (Table 2).

**Table 2:** Seropositivity inferences by binary and probabilistic classifiers

Inference	Binary classifier	Probabilistic classifier
Form of results for each individual *i*	*Yi* = 0, 1 if the individual is predicted to be positive or negative	0 ≤ *Pi* ≤ 1, the estimated probability an individual is positive or negative
Estimate seropositive fraction of sampled population	The mean of *Yi*	The mean of *Pi*

There are a plethora of supervised learning methods available. Some are based on standard statistical methods, such as logistic regression, while others attempt to define linear or nonlinear boundaries, such as support vector machines (SVM), which can be extended to return probabilities using methods such as Platt scaling. The linear methods are expected to produce a monotonic gradient across the plane defined by the two measures, yet others attempt to learn local classification rules which are unlikely to be monotonic.

We compared different approaches to 3 SD and 6 SD cutoffs (Supplementary figure 2a). Of the SD‐based boundaries (which are, by definition, composed of horizontal or vertical lines), the 6 SD anti‐spike IgG classifier perfectly separates the training data, but there are many control and test sample datapoints lying very close to the boundary (Figure 2a). We found that logistic regression learnt an almost identical boundary to Spike 6 SD. The other methods exploited both anti‐RBD and anti‐S measurements, predicting seropositivity when either anti‐S and anti‐RBD were high or when both were moderately high, although the shape of the decision boundaries varied, with linear discriminant analysis finding a narrow boundary, and SVM (linear or quadratic) a much wider region in which samples had uncertain classification. Interestingly, when we applied these methods, and equal‐weighted ensemble learners, to the test data from blood donors and pregnant women, we found much greater similarity between the different probabilistic methods than between the SD‐based approaches, and greater similarity between the probabilistic methods applied to augmented rather than observed data. Furthermore, the learners balanced the extreme sensitivity and specificity behaviors of 3 and 6 SD (Supplementary figure 2b), supporting that these tools provide more statistically sound estimates than do SD‐based methods for population seroprevalence studies.

**Figure 3:**
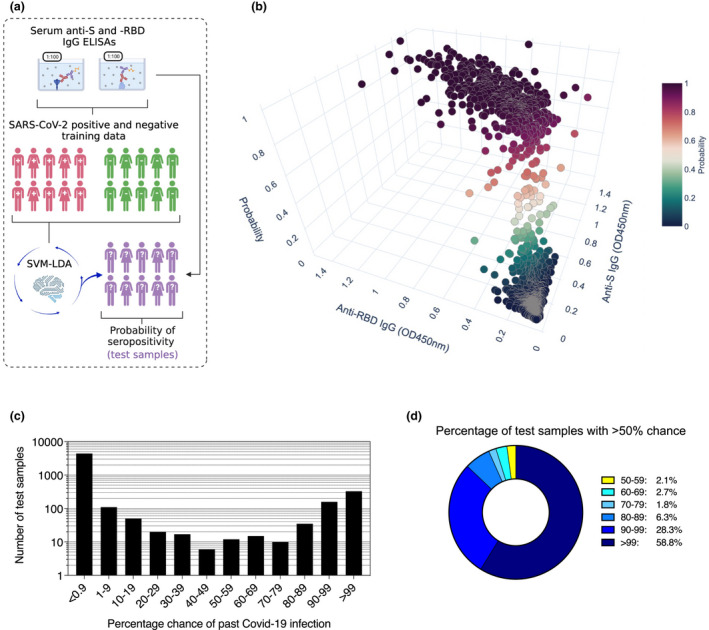
Likelihood of past SARS‐CoV‐2 infection at the individual level in blood donors and pregnant women test data. **(a)** Schematic representation of the probabilistic learner strategy for estimating probability of seropositivity. Training data consisted of *n* = 138 RT‐PCR cases and *n* = 595 pre‐pandemic negative controls. **(b)** Individual probability of past infection in blood donor and pregnant women test data (*n* = 5100) according to the SVM‐LDA learner. **(c)** Number of samples per % chance interval in the test data according to SVM‐LDA. **(d)** For test samples with > 50% chance of past infection, the proportion in different intervals according to SVM‐LDA.

For example, we applied the equal‐weighted ensemble learner of linear discriminant analysis and SVM (SVM‐LDA, Figure 3a and b) to our healthy donor population data. We chose to apply this learner as it appeared to combine the benefits of the parent methods, having an average sensitivity > 99.3% and specificity > 99.8%. In samples of unknown serostatus, the learner found *n* = 328 (6.4%) samples to have a > 99% chance of infection, while *n* = 4399 (86.3%) samples had < 0.9% chance (Figure 3c). A toal of 230 samples (4.5%) had between a 50 and 99% chance of being seropositive, while *n* = 203 (4.0%) samples had between a 1 and 49% chance. Amongst test samples with > 50% likelihood of past infection (*n* = 558), 58.8% had > 99% likelihood of seropositivity, 28.3% had between 90 and 99%, and 6.3% scored between 80 and 89% (Figure 3d). This contrasted with SD cutoffs, which identified *n* = 657 samples (12.9%) to be seropositive against both antigens at 3 SD, and *n* = 509 (10.0%) samples to remain so at 6 SD (Supplementary table 4). Therefore, when using a 6 SD cutoff compared to 3 SD, an additional 95 samples (1.9%) with a 2–41% chance of past infection, and 53 (1%) samples with a 50–97% chance, were classified as seronegative, further highlighting the need for closer scrutiny of values in the low end of the range.

## Discussion

Our ELISA data illustrated that all confirmed SARS‐CoV‐2 RT‐PCR+ individuals surveyed developed detectable antibodies against the ancestral SARS‐CoV‐2 spike glycoprotein, and mounted a neutralizing response capable of preventing S‐mediated cell entry, albeit at widely different titers. In addition, all but two of the healthy donor samples screened (*n = *56) had detectable neutralizing titers, although these were lower than in samples from confirmed infections. These data support that ancestral SARS‐CoV‐2 infection provoked a functional B cell response in the majority of those infected,^20^ and these data serve as a useful comparator to titers engendered by vaccination. In our study of adult RT‐PCR+ cases, conducted before the roll‐out of vaccines, disease severity was the primary driver of Ab levels. The first generation of COVID‐19 vaccines have been reported to generate neutralizing antibody titers comparable to those we observed in the samples from individuals who had mild (non‐hospitalized) infection, also from the Stockholm area.^11^, ^13^, ^16^

For epidemiological reasons, it is critical to be able to accurately determine the frequency of seropositivity in different population groups. However, this is complicated by differences in antibody levels between age groups,^26^, ^27^ for example, and low‐titer values, which in some cases – and increasingly with time since infection or vaccination^28^, ^29^ – can overlap outlier values amongst negative controls. Test samples with true low anti‐viral titers fall into the low‐end of the range, highlighting the need to better understand the assay boundary as the Ab response contracts. For example, improvements in this area could improve estimates of vaccine response duration, as well as seroprevalence. Indeed, we show that vaccine‐induced anti‐RBD titers can be difficult to classify within 3 months of the second vaccine dose in a subset of individuals.

Here, we propose that probabilistic approaches may be more suitable than SD‐based approaches to Ab testing because decisions are made on the basis of both positive and negative training data rather than thresholds based on negative training data alone, and they can infer information from multiple antigens simultaneously. We also emphasize, however, that evaluating optimal decision rules in a developing pandemic is complicated by the nature of training data available, which should be representative of the true range observed in the population. In our training data, 65% of the cohort was classified as having a mild (or non‐hospitalized) infection, while it is also possible that those who sought participation in research studies as hospital employees suspected a past infection, e.g. compared to an asymptomatic person. The numerous large cohorts collected during the pandemic serve as a useful resource, in this regard.

Such methods, and the implementation presented, provide a framework for the analysis of data from different assay platforms to assess immunity after SARS‐CoV‐2 infection or spike‐based vaccination. They also facilitate longitudinal studies to address the durability of immunity and comparisons of responses in different cohorts in relation to clinical features. Furthermore, although not intended as the main application here, such methods have the potential to communicate more nuanced information to individuals after an Ab test. If applied on an individual basis, the communication of probability needs to be approached with care in the clinical setting to ensure that what is described matches what an individual interprets.

## Methods

Study samples are defined in Table 1.

### Human samples and ethical declaration

Samples from SARS‐CoV‐2 RT‐PCR‐positive individuals and admitted COVID‐19 patients (*n* = 105) were collected by the attending clinicians and processed through the Departments of Medicine and Clinical Microbiology at the Karolinska University Hospital. Samples were used in accordance with approval by the Swedish Ethical Review Authority (registration no. 2020‐02811). All personal identifiers were pseudo‐anonymized, and all clinical feature data were blinded to the researchers carrying out experiments until data generation was complete. RT‐PCR testing for SARS‐CoV‐2 RNA was by upper respiratory tract sampling at Karolinska University Hospital and was carried out using different accredited RT‐PCR platforms. Although cycle threshold (Ct) values provide a semi‐quantitative measure, as Ct values were determined using different platforms, these could not be directly compared. SARS‐CoV‐2 RT‐PCR+ individuals (*n* = 105) were questioned about the date of symptom onset at their initial consultation and followed‐up for serology during their care, up to 2 months post‐diagnosis. Serum from SARS‐CoV‐2 RT‐PCR+ individuals was collected 6–63 days after diagnosis, with the median time from symptom onset to RT‐PCR testing being 5 days.

Hospital workers at Karolinska University Hospital were invited to test for the presence of SARS‐CoV‐2 RNA in throat swabs in April 2020 and virus‐specific IgG in serum in July 2020. We screened an additional *n = *33 RT‐PCR+ individuals to provide additional training data for machine learning approaches. All participants provided written informed consent. The study was approved by the National Ethical Review Agency of Sweden (2020‐01620) and the work was performed accordingly.

Sampling of individuals post‐vaccination was approved by the Swedish Ethics Review Authority (Dnr 2021‐00055). All individuals were included in the study after informed consent (*n* = 30). Ten individuals received two doses of BNT162b2 (Pfizer), 10 received two doses of mRNA‐1273 (Moderna), and 10 received two doses of ChAdOx‐1 (AstraZeneca).

Anonymized samples from blood donors (*n* = 100/week) and pregnant women (*n* = 100/week) were randomly selected by the department of Clinical Microbiology, Karolinska University Hospital. No metadata, such as age or sex information were available for these anonymized samples. Pregnant women were sampled as part of routine infectious diseases screening during the first trimester of pregnancy. Blood donors (*n* = 595) collected through the same channels a year previously were randomly selected for use as negative controls. Serum samples from individuals testing RT‐PCR+ for endemic coronaviruses (CoVs), 229E, HKU1, NL63, OC43 (*n* = 20, ECV+) in the prior 2–6 months, were used as additional negative controls during assay development. The use of study samples was approved by the Swedish Ethical Review Authority (registration no. 2020‐01807).

### Serum sample processing

Blood samples were collected by the attending clinical team and serum isolated by the Department of Clinical Microbiology following standard protocols. Samples were barcoded and stored at −20°C until use. Serum samples from vaccinated individuals were processed at Umeå Universitet as previously described,^30^ and stored at −80°C until use. Serum samples were not heat‐inactivated for ELISA protocols but were heat‐inactivated at 56°C for 60 min for *in vitro* neutralization experiments.

### SARS‐CoV‐2 antigen generation

The plasmid for expression of the SARS‐CoV‐2 prefusion‐stabilized spike ectodomain with a C‐terminal T4 fibritin trimerization motif was obtained from Wrapp *et al*.^17^ The plasmid was used to transiently transfect FreeStyle 293F cells using FreeStyle MAX reagent (Thermo Fisher Scientific). The ectodomain was purified from filtered supernatant on Streptactin XT resin (IBA Lifesciences), followed by size‐exclusion chromatography on a Superdex 200 in 5 mM Tris pH 8, 200 mM NaCl.

The RBD domain (RVQ – QFG) of SARS‐CoV‐2 was cloned upstream of a Sortase A recognition site (LPETG) and a 6xHIS tag, and expressed in 293F cells as described above. RBD‐HIS was purified from filtered supernatant on His‐Pur Ni‐NTA resin (Thermo Fisher Scientific), followed by size‐exclusion chromatography on a Superdex 200. The nucleocapsid was purchased from Sino Biological and was not used beyond assay development.

### Anti‐SARS‐CoV‐2 ELISA

96‐well ELISA plates (Nunc MaxiSorp) were coated with SARS‐CoV‐2 S trimers, RBD or nucleocapsid (100 ng per well) in PBS overnight at 4°C. Plates were washed six times with PBS‐Tween‐20 (0.05%) and blocked using PBS‐5% no‐fat milk. Human serum samples were thawed at room temperature, diluted (1:100 unless otherwise indicated), and incubated in blocking buffer for 1 h (with vortexing) before plating. Serum samples were incubated overnight at 4°C before washing, as before. Secondary HRP‐conjugated anti‐human antibodies were diluted in blocking buffer and incubated with samples for 1 h at room temperature. Plates were washed a final time before development with TMB Stabilized Chromogen (Invitrogen). The reaction was stopped using 1 m sulphuric acid and optical density (OD) values were measured at 450 nm using an Asys Expert 96 ELISA reader (Biochrom Ltd.). Secondary antibodies (all from Southern Biotech) and dilutions used: goat anti‐human IgG (2014–05) at 1:10 000; goat anti‐human IgM (2020–05) at 1:1000; goat anti‐human IgA (2050–05) at 1:6000. All assays of the same antigen and isotype were developed for their fixed time and samples were randomized and run together on the same day when comparing binding between RT‐PCR+ individuals. Negative control samples were run alongside test samples in all assays and raw data were log transformed for statistical analyses.

Our S and RBD ELISA assays had the following sensitivity & specificity at fixed SD thresholds:

Spike 3SD: 100% (95% CI [97.5–100.0]) & 99.0% (95% CI [98.6–99.0]).
Spike 6SD: 100% (95% CI [97.5–100.0]) & 99.9% (95% CI [99.6–100.0]).
RBD 3SD: 100% (95% CI [97.5–100.0]) & 99.0% (95% CI [98.4–99.4]).
RBD 6SD: 98.0% (95% CI [94.2–99.3]) & 99.9% (95% CI [99.6–100.0]).

### *In vitro* virus neutralization assay

Pseudotyped viruses were generated by the co‐transfection of HEK293T cells with plasmids encoding the SARS‐CoV‐2 spike protein harboring an 18 amino acid truncation of the cytoplasmic tail^17^; a plasmid encoding firefly luciferase; a lentiviral packaging plasmid (8455, Addgene) using Lipofectamine 3000 (Invitrogen). Media was changed 12–16 h post‐transfection and pseudotyped viruses harvested at 48 and 72 h, filtered through a 0.45 µm filter and stored at −80°C until use. Pseudotyped neutralization assays were adapted from protocols validated to characterize the neutralization of HIV, but with the use of HEK293T‐ACE2 cells. Briefly, pseudotyped viruses sufficient to generate ~100 000 RLUs were incubated with serial dilutions of heat‐inactivated serum for 60 min at 37°C. Approximately 15 000 HEK293T‐ACE2 cells were then added to each well and the plates incubated at 37°C for 48 h. Luminescence was measured using Bright‐Glo (Promega) according to the manufacturer’s instructions on a GM‐2000 luminometer (Promega) with an integration time of 0.3 s. The limit of detection was at a 1:45 serum dilution.

### IL‐6 cytometric bead array

Serum IL‐6 levels were measured in a subset of RT‐PCR+ serum samples (*n* = 64) using an enhanced sensitivity cytometric bead array against human IL‐6 from BD Biosciences. Protocols were carried out according to the manufacturer’s recommendations and data acquired using a BD Celesta flow cytometer.

### Statistical analysis of SARS‐CoV‐2 RT‐PCR+ data

All univariate comparisons were performed using non‐parametric analyses (Kruskal‐Wallis, stratified Mann‐Whitney, hypergeometric exact tests and Spearman rank correlation) as indicated, while multivariate comparisons were performed using linear regression of log‐transformed measures and Wald tests. For multivariate tests, all biochemical measures (IL‐6, PSV ID50 neut., IgG, IgA, IgM) were log‐transformed to improve the symmetry of the distribution. As “days since first symptom” and ”days since RT‐PCR+ test” were highly correlated, we did not include both in any single analysis. Instead, we show results for one, then the other (Supplementary table 2).

### Probabilistic algorithms for classifying antibody positivity

Prior to analysis, each sample OD was standardized by dividing by the mean OD of “no sample controls” on that plate or other plates run on the same day. This resulted in more similar distributions for 2019 blood donor samples with 2020 blood donors and pregnant women, as well as smaller coefficients of variation amongst RT‐PCR+ COVID patients for both Spike and RBD.

Our probabilistic learning approach consisted of evaluating different algorithms suited to ELISA data, which we compared through ten‐fold cross‐validation: logistic regression (LOG), linear discriminant analysis (LDA), support vector machines (SVM) with a linear kernel, and quadratic SVM (SVM2). Logistic regression models the log odds of a sample being seropositive as a linear equation with a resulting linear decision boundary, while LDA finds a linear boundary by maximizing the ratio of the within‐group to between‐group sum of squares. When applied to new data, both logistic regression and LDA can provide an estimate of the probability of each new sample being seropositive. Support vector machines (SVM) is an altogether different approach that constructs a boundary that maximally separates the classes (i.e. the margin between the closest member of any class and the boundary is as wide as possible), hence points lying far away from their respective class boundaries do not play an important role in shaping it. SVM thus puts more weight on points closest to the class boundary, which in our case is far from being clear. Linear SVM has one tuning parameter C, a cost, with larger values resulting in narrower margins. We tuned C on a vector of values (0.001, 0.01, 0.5, 1, 2, 5, 10) via an internal 5‐fold cross validation with 5 repeats (with the winning parameter used for the final model for the main cross validation iteration). We also note that the natural output of SVM are class labels rather than class probabilities, so the latter are obtained via the method of Platt.^31^

We evaluated these methods using cross‐validation, according to three strategies: i) random: individuals were sampled into folds at random, ii) stratified: individuals were sampled into folds at random, subject to ensuring the balance of cases: controls remained fixed and iii) unbalanced: individuals were sampled into folds such that each fold was deliberately skewed to under or over‐represent cases compared to the total sample. We sought a method with performance that was consistently good across all cross‐validation sampling schemes, because the true proportion of cases in the test data is unknown, and we want a method that is not overly sensitive to the proportion of cases in the training data. We chose to assess performance using sensitivity and specificity, as well as consistency.

Given the good performance of all learners, we considered the prediction surface associated with each SVM, LDA, SVM‐LDA ensemble, and the standard 3‐SD, 6‐SD hard decision boundaries. Note that while methods trained on both proteins can draw decision contours at any angle, SD methods are limited to vertical or horizontal lines. We can see that success, or failure, of the SD cut‐offs depends on how many positive and negative cases overlap for a given measure (S or RBD) in the training sample. In the training data, the two classes are nearly linearly separable when each protein is considered on its own, which explains good performance of 3‐SD and 6‐SD thresholds. However, the test data contain many more points in the mid‐range of S‐RBD, in line with milder infections in most adults, which makes hard cut‐offs a problematic choice for classifying test samples.

We trained the learners on all 733 training samples and used these to predict the probability of anti‐SARS‐CoV‐2 IgG in blood donors and pregnant volunteers sampled in 2020‐2021. We inferred the proportion of the sampled population with positive antibody status each week using multiple imputation. We repeatedly (1000 times) imputed antibody status for each individual randomly according to the ensemble prediction, and then analyzed each of the 1000 datasets in parallel, combining inference using Rubin's rules, derived for the Wilson binomial proportion confidence interval.^32^ To compute confidence intervals for sensitivity and specificity, we dichotomized predictions of seropositivity at *P* > 0.5 or ≤ 0.5 and computed average sensitivity, specificity, and 95% confidence intervals for each fold in the cross validation via Wilson's method before averaging over all folds.

## Conflict of interest

The study authors declare no competing financial interests that could compromise the study. CW also receives funding from GlaxoSmithKline and Merck Sharp & Dohme; these funders had no role in the design, analysis or interpretation of this study. The views expressed are those of the authors.

## Author contributions

**Xaquin Castro Dopico:** Conceptualization; Formal Analysis; Investigation; Writing – original draft. **Sandra Muschiol:** Resources. **Nastasiya F Grinberg:** Formal analysis; Methodology. **Soo Aleman:** Resources. **Daniel J Sheward:** Investigation. **Leo Hanke:** Resources. **Marcus Ahl:** Resources. **Linnea Vikström:** Resources. **Mattias NE Forsell:** Funding acquisition; Resources. **Jonathan Coquet:** Investigation. **Gerald McInerney:** Funding acquisition; Resources. **Joakim Dillner:** Resources. **Gordana Bodganovic:** Resources. **Ben Murrell:** Funding acquisition; Methodology. **Jan Albert:** Resources; Supervision. **Chris Wallace:** Formal analysis; Funding acquisition; Investigation; Methodology; Software; Writing ‐ reviewing/editing of final draft. **Gunilla B Karlsson Hedestam:** Conceptualization; Funding acquisition; Investigation; Supervision; Writing ‐ reviewing/editing of final draft.

## Supporting information

Supplementary figures 1‐2

Supplementary tables 1–4

## Data Availability

Data generated as part of the study, along with code for implementation, is openly available in via our GitHub repository: https://github.com/chr1swallace/elisa‐paper.

## References

[1] Murhekar MV , Clapham H . COVID‐19 serosurveys for public health decision making. Lancet Glob Health 2021; 9: e559–e560. 33705691 10.1016/S2214-109X(21)00057-7 PMC8049585

[2] GeurtsvanKessel CH , Okba NMA , Igloi Z *et al*. An evaluation of COVID‐19 serological assays informs future diagnostics and exposure assessment. Nat Commun 2020; 11: 3436. doi:10.1038/s41467-020-17317-y 32632160 PMC7338506

[3] Rostami A , Sepidarkish M , Leeflang MMG *et al*. SARS‐CoV‐2 seroprevalence worldwide: a systematic review and meta‐analysis. Clin Microbiol Infect 2021; 27: 331–340. 33228974 10.1016/j.cmi.2020.10.020 PMC7584920

[4] Kohmer N , Westhaus S , Rühl C , Ciesek S , Rabenau H . Clinical performance of different SARS‐CoV‐2 IgG antibody tests. J Med Virol 2020; 92: 2243–2247. 32510168 10.1002/jmv.26145 PMC7300776

[5] Cervia C , Nilsson J , Zurbuchen Y . Systemic and mucosal antibody responses specific to SARS‐CoV‐2 during mild versus severe COVID‐19. J Allergy Clin Immunol. 2021; 147: 545–557. 33221383 10.1016/j.jaci.2020.10.040 PMC7677074

[6] Shrock E , Fujimura E , Kula T *et al*. Viral epitope profiling of COVID‐19 patients reveals cross‐reactivity and correlates of severity. Science 2020; 270: eabd4250. 10.1126/science.abd4250 PMC7857405 32994364

[7] Marklund E , Leach S , Axelsson H *et al*. Serum‐IgG responses to SARS‐CoV‐2 after mild and severe COVID‐19 infection and analysis of IgG non‐responders. PLoS One 2020; 15: e0241104. 33085715 10.1371/journal.pone.0241104 PMC7577439

[8] Wang Y , Zhang LU , Sang L *et al*. Kinetics of viral load and antibody response in relation to COVID‐19 severity. J Clin Invest 2020; 130: 5235–5244. 32634129 10.1172/JCI138759 PMC7524490

[9] Castro Dopico X , Ols S , Loré K , Karlsson Hedestam GB . Immunity to SARS‐CoV‐2 induced by infection or vaccination. J Intern Med 2022; 291: 32–50. 34352148 10.1111/joim.13372 PMC8447342

[10] Wang Z , Schmidt F , Weisblum Y *et al*. mRNA vaccine‐elicited antibodies to SARS‐CoV‐2 and circulating variants. Nature 2021; 592: 616–622. 33567448 10.1038/s41586-021-03324-6 PMC8503938

[11] Mulligan MJ , Lyke KE , Kitchin N *et al*. Phase I/II study of COVID‐19 RNA vaccine BNT162b1 in adults. Nature 2020; 586: 589–593. 32785213 10.1038/s41586-020-2639-4

[12] Khoury DS , Cromer D , Reynaldi A *et al*. Neutralizing antibody levels are highly predictive of immune protection from symptomatic SARS‐CoV‐2 infection. Nat Med 2021; 27: 1205–1211. 34002089 10.1038/s41591-021-01377-8

[13] Castro Dopico X , Muschiol S , Christian M *et al*. Seropositivity in blood donors and pregnant women during the first year of SARS‐CoV‐2 transmission in Stockholm, Sweden. J Intern Med 2021; 290: 666–676. 34008203 10.1111/joim.13304 PMC8242905

[14] Gudbjartsson DF , Norddahl GL , Melsted P *et al*. Humoral immune response to SARS‐CoV‐2 in Iceland. N Engl J Med 2020; 383: 1724–1734. 32871063 10.1056/NEJMoa2026116 PMC7494247

[15] Vanshylla K , Di Cristanziano V , Kleipass F *et al*. Kinetics and correlates of the neutralizing antibody response to SARS‐CoV‐2 infection in humans. Cell Host Microbe 2021; 29: 917–929.e4. 33984285 10.1016/j.chom.2021.04.015 PMC8090990

[16] Jackson LA , Anderson EJ , Rouphael NG *et al*. An mRNA vaccine against SARS‐CoV‐2 — preliminary report. N Engl J Med 2020; 383: 1920–1931. 32663912 10.1056/NEJMoa2022483 PMC7377258

[17] Wrapp D , Wang N , Corbett KS *et al*. Cryo‐EM structure of the 2019‐nCoV spike in the prefusion conformation. Science 2020; 367: 1260–1263. 32075877 10.1126/science.abb2507 PMC7164637

[18] Hanke L , Vidakovics Perez L , Sheward DJ *et al*. An alpaca nanobody neutralizes SARS‐CoV‐2 by blocking receptor interaction. Nat Commun 2020; 11: 4420. 32887876 10.1038/s41467-020-18174-5 PMC7473855

[19] Long Q‐X , Liu B‐Z , Deng H‐J *et al*. Antibody responses to SARS‐CoV‐2 in patients with COVID‐19. Nat Med 2020; 26: 845–848. 32350462 10.1038/s41591-020-0897-1

[20] Gudbjartsson DF , Helgason A , Jonsson H *et al*. Spread of SARS‐CoV‐2 in the Icelandic population. N Engl J Med 2020; 382: 2302–2315. 32289214 10.1056/NEJMoa2006100 PMC7175425

[21] Gaebler C , Wang Z , Lorenzi JCC *et al*. Evolution of antibody immunity to SARS‐CoV‐2. Nature 2021; 591: 639–644. 33461210 10.1038/s41586-021-03207-w PMC8221082

[22] Mandolesi M , Sheward DJ , Hanke L *et al*. SARS‐CoV‐2 protein subunit vaccination of mice and rhesus macaques elicits potent and durable neutralizing antibody responses. Cell Reports Med 2021; 2: 100252. 10.1016/j.xcrm.2021.100252 PMC8020888 33842900

[23] Favresse J , Bayart J‐L , Mullier F *et al*. Antibody titres decline 3‐month post‐vaccination with BNT162b2. Emerg Microbes Infect 2021; 10: 1495–1498. 34232116 10.1080/22221751.2021.1953403 PMC8300930

[24] Erice A , Varillas‐Delgado D , Caballero C . Decline of antibody titres three months after two doses of BNT162b2 in non‐immunocompromised adults. Clin Microbiol Infect 2022; 28: e1–139.e4. 10.1016/j.cmi.2021.08.023 PMC8426320 34508885

[25] Israel A , Shenhar Y , Green I *et al*. Large‐scale study of antibody titer decay following BNT162b2 mRNA vaccine or SARS‐CoV‐2 infection. Vaccines 2021; 10: 64. 35062724 10.3390/vaccines10010064 PMC8781423

[26] Tosif S , Neeland MR , Sutton P *et al*. Immune responses to SARS‐CoV‐2 in three children of parents with symptomatic COVID‐19. Nat Commun 2020; 11: 5703. 33177504 10.1038/s41467-020-19545-8 PMC7658256

[27] Weisberg SP , Connors TJ , Zhu Y *et al*. Distinct antibody responses to SARS‐CoV‐2 in children and adults across the COVID‐19 clinical spectrum. Nat Immunol 2021; 22: 25–31. 33154590 10.1038/s41590-020-00826-9 PMC8136619

[28] Seow J , Graham C , Merrick B *et al*. Longitudinal observation and decline of neutralizing antibody responses in the three months following SARS‐CoV‐2 infection in humans. Nat Microbiol 2020; 5: 1598–1607. 33106674 10.1038/s41564-020-00813-8 PMC7610833

[29] Long Q‐X , Tang X‐J , Shi Q‐L *et al*. Clinical and immunological assessment of asymptomatic SARS‐CoV‐2 infections. Nat Med 2020; 26: 1200–1204. 32555424 10.1038/s41591-020-0965-6

[30] Normark J , Vikström L , Gwon Y‐D *et al*. Heterologous ChAdOx1 nCoV‐19 and mRNA‐1273 vaccination. N Engl J Med 2021; 385: 1049–1051. 34260850 10.1056/NEJMc2110716 PMC8314734

[31] Platt J . Probabilistic outputs for support vector machines and comparisons to regularized likelihood methods. Adv Large Margin Classif 1999; 10: 61–74.

[32] Lott A , Reiter JP . Wilson confidence intervals for binomial proportions with multiple imputation for missing data. Am Stat 2020; 74: 109–115.

