# Supplementary material for: Probabilistic classification of anti‐SARS‐CoV‐2 antibody responses improves seroprevalence estimates
Source: Clin Transl Immunology. 2022 Mar 2;11(3):e1379. doi: 10.1002/cti2.1379 (PMC8891432; doi:10.1002/cti2.1379)
Supplement: Supplementary file 1 — Supplementary figures 1‐2 [file CTI2-11-e1379-s001.pdf]

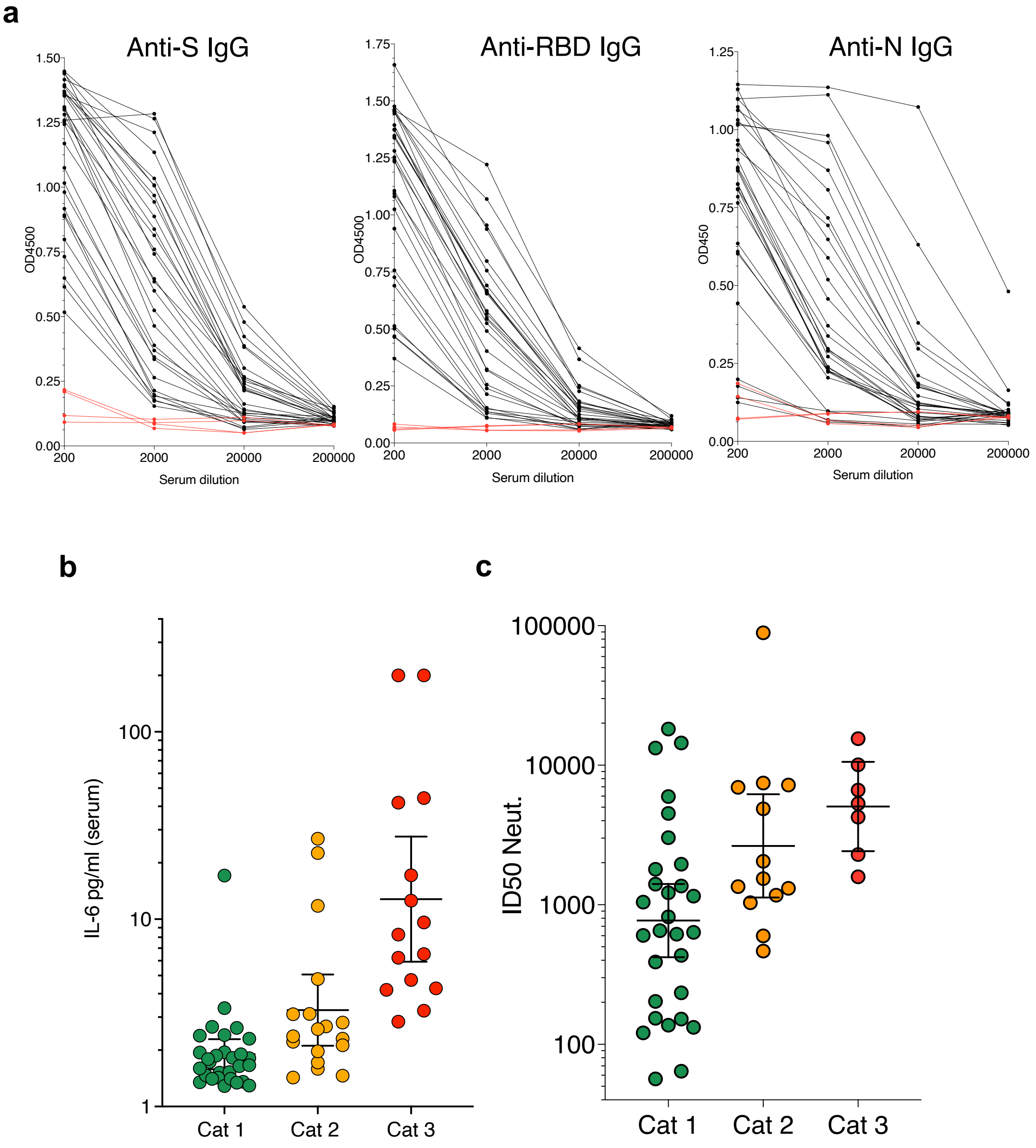

Supplementary Figure 1: Assay development and COVID-19 antibody phenotypes.

(**a**) Serial dilution of IgG responses so S, RBD and N in a random subset of RT-PCR+ samples used for assay development. Indiviuduals PCR+ for endemic coronaviruses are shown by red dots and lines for each assay. Anti-S Ab responses were frequently detected at 1:20,000 serum dilution using this assay. All samples for the study were run at 1:100 dilution. (**b**) Serum IL-6 levels in a random subset of RT-PCR+ samples across the clinical spectrum. IL-6 levels were measured using a cytometric bead array. (**c**) *In vitro* virus neutralizing responses in a random subset of RT-PCR+ samples across the clinical spectrum. *Error bars represent the geometric mean with 95% CIs.*

Figure S1

a

b

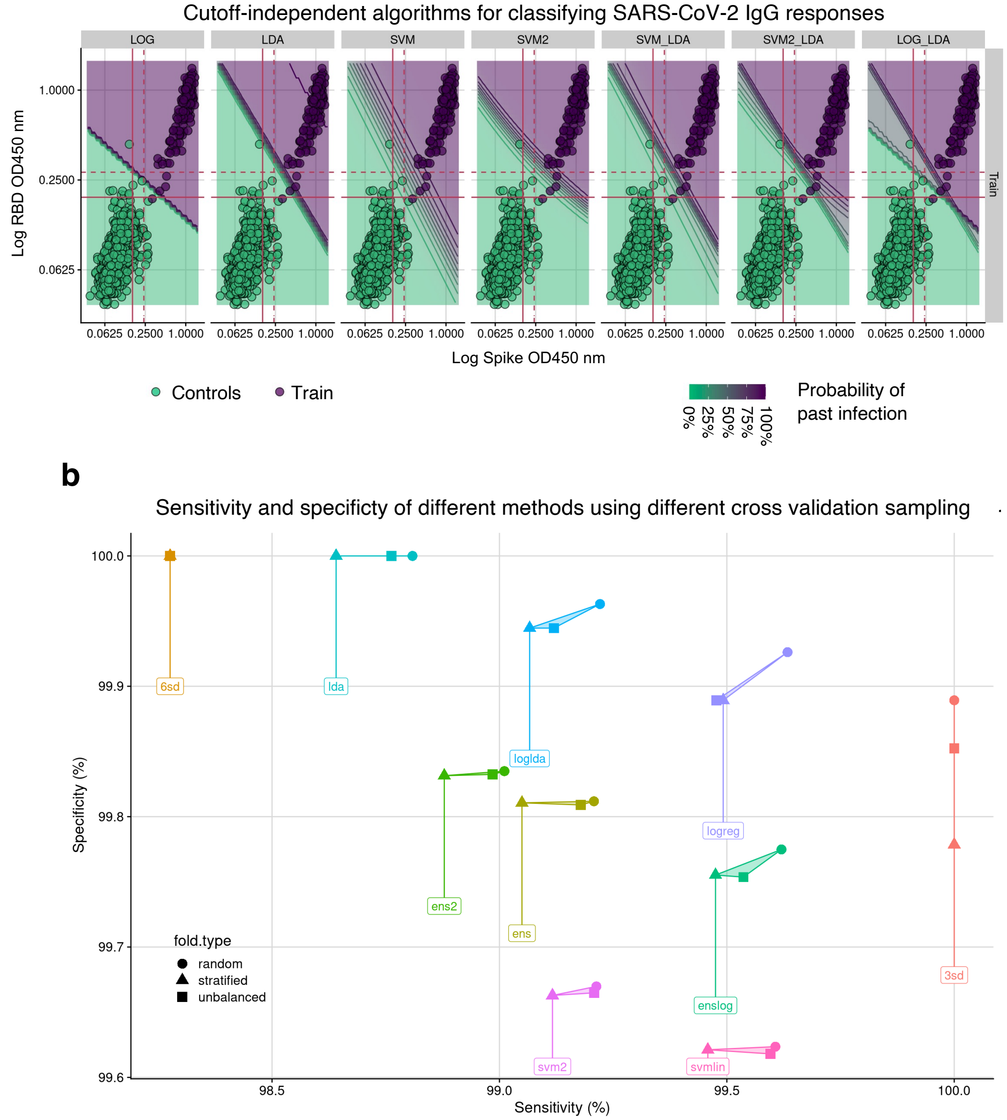

Supplementary Figure 2: Comparison of probabilistic and SD-based methods for determining seropositivity.

(**a**) Comparison of logistic regression (LOG), linear discriminant analysis (LDA), support vector machines (SVM) and quadratic SVM (SVM2) to 3 and 6 SD cutoffs in the training data. Ensemble learners that provide equal-weighted outputs from their parent methods were also created: ens: SVM-LDA; ens2: SVM2-LDA. (**b**) Sensitivity and specificity behaviors of each method.

Figure S2
